# Singing emotionally: a study of pre-production, production, and post-production facial expressions

**DOI:** 10.3389/fpsyg.2014.00262

**Published:** 2014-04-29

**Authors:** Lena R. Quinto, William F. Thompson, Christian Kroos, Caroline Palmer

**Affiliations:** ^1^Department of Psychology, Macquarie UniversitySydney, NSW, Australia; ^2^Department of Psychology, McGill UniversityMontreal, QC, Canada

**Keywords:** singing, emotional communication, point-light displays, face motion

## Abstract

Singing involves vocal production accompanied by a dynamic and meaningful use of facial expressions, which may serve as ancillary gestures that complement, disambiguate, or reinforce the acoustic signal. In this investigation, we examined the use of facial movements to communicate emotion, focusing on movements arising in three epochs: before vocalization (pre-production), during vocalization (production), and immediately after vocalization (post-production). The stimuli were recordings of seven vocalists' facial movements as they sang short (14 syllable) melodic phrases with the intention of communicating happiness, sadness, irritation, or no emotion. Facial movements were presented as point-light displays to 16 observers who judged the emotion conveyed. Experiment 1 revealed that the accuracy of emotional judgment varied with singer, emotion, and epoch. Accuracy was highest in the production epoch, however, happiness was well communicated in the pre-production epoch. In Experiment 2, observers judged point-light displays of exaggerated movements. The ratings suggested that the extent of facial and head movements was largely perceived as a gauge of emotional arousal. In Experiment 3, observers rated point-light displays of scrambled movements. Configural information was removed in these stimuli but velocity and acceleration were retained. Exaggerated scrambled movements were likely to be associated with happiness or irritation whereas unexaggerated scrambled movements were more likely to be identified as “neutral.” An analysis of singers' facial movements revealed systematic changes as a function of the emotional intentions of singers. The findings confirm the central role of facial expressions in vocal emotional communication, and highlight individual differences between singers in the amount and intelligibility of facial movements made before, during, and after vocalization.

## Introduction

Emotional communication has been investigated in many different modalities including facial expressions (Elfenbein and Ambady, 2002), tone of voice (Johnstone and Scherer, 2000), music (Juslin and Laukka, 2003; Gabrielsson and Lindström, 2010), and gestures associated with music performance (Davidson, 1993; Thompson et al., 2005; Vines et al., 2006). Perceivers are sensitive to the information contained in these channels of communication and can decode emotional signals produced by individuals within and across cultures (Russell et al., 2003; Thompson and Balkwill, 2010).

In music, emotions are encoded in a range of acoustic attributes, including contour, modality, pitch height, intensity, tempo, and rhythm (for a review, see Juslin and Sloboda, 2010). Music performers often supplement these attributes with visual signals of emotion to enhance the clarity or impact of emotional communication. The facial expressions and gestures of performers are known to influence the perception of expressiveness (Davidson, 1993, 1995), tension (Vines et al., 2006), timbre (Saldaña and Rosenblum, 1993), dissonance (Thompson et al., 2005), note duration (Schutz and Lipscomb, 2007), interval size (Thompson and Russo, 2007), phrase structure (Ceaser et al., 2009), and emotion (Dahl and Friberg, 2007; Thompson et al., 2008). Ensemble musicians also use gestures and eye contact to facilitate coordinated action, particularly in sections that introduce new or important material (Williamon and Davidson, 2002).

Studies that have used video recordings have demonstrated that facial expressions can communicate a range of information associated with music performance. Facial expressions used in guitar performances by B.B. King, for example, appear to signal technical difficulty whereas other facial expressions appear to reflect current levels of dissonance associated with a musical passage (Thompson et al., 2005). A case study of the pianist Lang Lang revealed that his facial expressions closely mirrored the musical structure and the underlying meaning of a programmatic musical work (Davidson, 2012). Wöllner (2008) found that expressiveness ratings for audio-visual presentations of orchestral music were more closely correlated with ratings of the conductor's facial expressions than with ratings of the conductor's arms or blurred body movements. Similarly, if auditory information is held constant across renditions but paired with different visual gestures, performance judgments differ (Behne and Wöllner, 2011). A recent meta-analysis revealed a moderate but reliable effect size of the visual domain on perceptions of expressiveness, overall quality, and liking (Platz and Kopiez, 2012).

Musicians can also communicate discrete emotional states such as “happy” and “sad” through the use of facial expressions (Thompson et al., 2005). A sounded major third is judged to be sadder when combined with facial expressions made while singing a minor third, and a sounded minor third is judged to be happier when combined with facial expressions made while singing a major third (Thompson et al., 2008). Dahl and Friberg (2007) found that the emotional intentions of happiness, sadness, and anger were communicated well by the body and head movements of musicians, such that viewers did not even need auditory information to determine the intended emotion.

Music performances are inherently dynamic and emotional responses may change over time (Schubert, 2004). Early work was largely restricted to examinations of static images. Examining the visual information available from complex dynamic motion in facial expressions and body movements was a challenge, particularly in isolating the core dynamic features that were used by perceivers to decode emotion. One method used to examine the contribution of motion to perception was through the use of point-light displays (PLDs). PLDs present the visual information in a reduced form. Before motion capture technology was developed, PLDs were achieved by placing reflective or white markers on dark clothing or a face that had been darkened with make-up. In this method, the form information from a single static image is difficult to identify and unique features are often lost. The addition of dynamic information allows viewers to easily identify biological motion (Blake and Shiffrar, 2007). Using PLDs, participants are able to decode emotion from facial expressions (Bassili, 1978), and even through the gait of point-light walkers (Halovic and Kroos, 2009). Participants are also better able to identify musicians' expressive intentions when presented with the body movements of performers (no sound) than when presented with the sounded performance without visual information (Davidson, 1993). Currently, motion capture allows researchers to record movement, quantitively analyse this movement, and develop PLD videos. Motion capture also allows for the manipulation of features in the point-light display (e.g., only showing particular features or developing non-biological control stimuli). A second method to understand the influence of movement on viewers' perception is to use full-video recordings. Full-video has often been used to examine the visual influence in music. To understand the specific features of interest, researchers sometimes occlude parts of the performer (e.g., Dahl and Friberg, 2007; Thompson et al., 2010) or use filtering methods so that specific features are difficult to identify (e.g., Wöllner, 2008).

Humans appear to be extremely sensitive to motion and emotional information such that the full apex of an emotional expression is not needed to decode emotion. Fiorentini et al. (2012) showed participants images of emotional expressions that developed over time and found that viewers perceived emotions well before the full emotional configuration was reached. One interpretation of these findings is that viewers make use of individual features that emerge early in the formation of a facial expression, such as lip and eyebrow movements. Such features are then used to make probabilistic judgments of an intended emotion.

In music, facial expressions and gestures often occur outside the boundaries of sounded music, for example, in moments of silence that occur before and after musical phrases are vocalized. These ancillary gestures are not a direct consequence of the physical constraints of vocal production but, rather, act to signal emotional, social, and other communicative goals (Davidson, 1995; Palmer, 2012). In some cases, facial expressions reinforce communicative goals that may be ambiguous in the sounded performance, clarifying the structural or emotional characteristics of the music.

Supporting this idea, Livingstone et al. (2009) reported that singers exhibited emotional facial expressions well before they were expected to sing. Musicians watched a model singer express a musical phrase communicating happiness, sadness, or no expression. They were then asked to sing back this phrase and their movements were recorded with motion capture. The results showed that musicians surrounded their vocalizations with meaningful facial expressions. Intended emotions were reflected in facial expressions before, during, and after vocalizations. These findings suggest that musicians hint at the emotional information that is forthcoming in a musical phrase, and sustain those emotional expressions after the cessation of that phrase. Such supra-production expressions may benefit audience members by optimizing their capacity to extract communicative intentions (see also Wanderley et al., 2005).

We used motion capture to examine the facial expressions of seven musicians as they sang phrases with each of four emotional intentions: happiness, sadness, irritation, and no emotion. Irritation was used instead of anger to convey a subtler version of the latter emotion. Facial expressions were captured and analyzed in three epochs: before the musicians began singing (pre-production), during singing (production), and once they had completed singing (post-production). Point-light displays of these facial expressions (without sound) were then presented to independent perceivers who judged their emotional content in the first experiment. In subsequent experiments, we presented the same facial movements to participants along with exaggerated forms (facial movements were algorithmically manipulated to contain a larger range of movements) and in scrambled forms (randomized the direction of marker movements, keeping range of motion constant). The scrambled condition showed the initial marker positions but as the motion started, the direction of the marker trajectory was randomly determined while keeping the range, velocity and acceleration constant. These manipulations allowed us to better understand the nature of the cues used by perceivers to decode emotional intentions.

## Experiment 1

The goal of Experiment 1 was to examine the ability of perceivers to decode the emotional dynamic facial expressions and head movements observed in point-light displays of seven singers. We expected that emotional decoding would be highest in the production phase, when musicians are most likely to be focusing on their communicative intentions. Although musicians may be more focused on communicating the emotion in the production phase, production constraints associated with singing might limit the capacity of singers to express emotion through movements of the mouth. The findings of Livingstone et al. (2009) suggest that the pre- and post-production epochs contain important movement information that singers use to communicate emotion through facial expressions made before and after singing. Perceivers appear to mimic the emotional expressions of singers (see also Chan et al., 2013) but it is unclear whether perceivers can use this information to accurately decode the intended emotion based solely on the motion information conveyed in point-light displays.

It was expected that some emotions would be better decoded depending on the epoch. For example, Bassili (1979), who used PLDs, found that anger is communicated through eyebrow movements and frowns, whereas happiness is communicated through mouth movements (which presumably do not occur in pre- and post-singing epochs). A study of singing using full-video found that happiness was not well communicated during singing, in contrast anger and sadness were communicated during singing (Scotto di Carlo and Guaitella, 2004). Thus, it was expected that the emotion of happiness may not be as well communicated in the production epoch as in the pre- or post-production epochs. In contrast, irritation and sadness were expected to be decoded equally well in each of the epochs.

Finally, we also expected individual differences between singers in their ability to communicate specific emotions, and their tendency to express emotions in facial expressions before and after vocalizations. Although emotional encoding and decoding occurs universally in static facial expressions (Ekman and Friesen, 1971), social norms influence the expression of certain emotions (Scherer et al., 2003) and there are individual differences in the ability to communicate emotionally in music (Davidson, 1993, 2012; Juslin, 2000; Wanderley et al., 2005; Dahl and Friberg, 2007; Timmers and Ashley, 2007). Wanderley et al. (2005) observed that clarinettists differed from each other in the use of idiosyncratic gestures such as knee bending, vertical shoulder movement, and circular movements of the clarinet bell. Similarly, Davidson (2012) observed variability in the body movements used by flautists and clarinettists. Despite such individual differences in performance gestures, perceivers are still able to decode emotional intentions. Consistent with Brunswik's lens model (1956; see also Juslin, 2000), emotional decoding is possible because there are several redundant cues associated with any one emotion, and perceivers evaluate such emotional cues probabilistically. A probabilistic decoding strategy allows perceivers to adapt to idiosyncratic strategies of communicating emotion. In the current study, while all singers were trained musicians, some had more experience as singers whereas others had more experience as instrumentalists. As such, we examined the ability of perceivers to decode emotional facial expressions for each singer separately.

### Methods

#### Musicians

Seven singers participated in the motion capture session. They were recruited through advertisements to local music theatre groups, drama societies, and choirs. Singers were selected on the following basis: (a) they were actively involved in music-making, (b) they were able to use facial expressions to communicate emotion, and (c) they were able to sing the melody in tune. Two judges determined whether an individual was a possible candidate for the session: One judge was a recording engineer with experience in music education and made decisions regarding the quality of the auditory information. The other judge was a researcher with experience in facial expressions and determined the quality of information conveyed through the visual domain.

All singers were currently involved in music. Most had been singing since childhood and had received extensive musical training. They had an average age of 29 years (*SD* = 12.64); an average of 9.83 (*SD* = 6.73; range = 3–20) years of formal music training; and an average of 22.83 (*SD* = 11.39; range = 5–45) years of active involvement in music. All were paid for their participation.

#### Motion capture equipment

**Figure 1:**
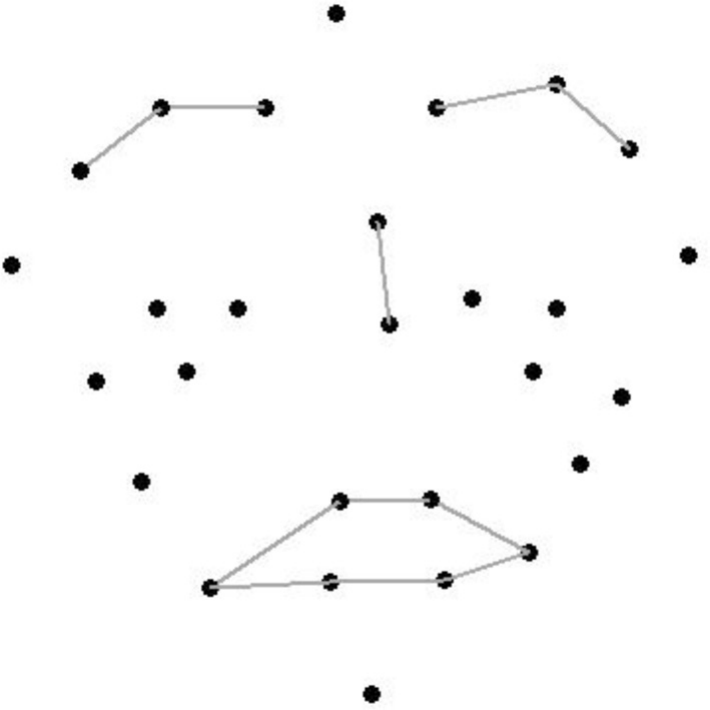
**The position of the markers outlining the major features of the face; lines indicate eyebrows, nose, and lips**.

Figure 1 illustrates the facial positions of 28 of the 29 Vicon markers that were placed on musicians using double-sided hypoallergenic tape. The musicians were asked to wear dark clothing and to avoid wearing make-up or sunscreen for the experimental session. Three markers were positioned on each eyebrow, two were positioned under each eye, six outlined the lips and three outlined the cheeks. One marker was placed on each of the following: chin, forehead, left and right temple, tip of the nose, nasion, and the shoulder as a reference point. The marker on the shoulder was excluded from the animated stimuli. The markers on the temples, shoulder and forehead were 9 mm in diameter and the remaining markers were 4 mm in diameter. The musicians were recorded with eight Vicon MX+ infrared cameras at a frame rate of 200 frames per second. Musicians stood in the middle of an 8-foot capture space (surrounded by the eight cameras).

#### Stimulus materials

**Figure 2:**
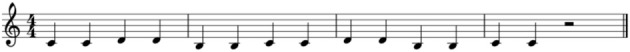
**The melody sung by performers**.

Singers were asked to sing the text phrase to an experimental melody (Figure 2) that was presented to them through headphones in a piano timbre. This melody was neutral with respect to its musical mode, which is known to influence emotional judgments (e.g., Hevner, 1935), and was synchronized to a metronome at a tempo of 500 ms per beat. Singers were instructed to sing one syllable of the scripted phrase on each beat.

Four text phrases were created, designed to be semantically neutral or ambiguous in terms of their emotional connotation (“The orange cat sat on a mat and ate a big, fat rat,” “The girl and boy walked to the fridge to fetch some milk for lunch,” “The broom is in the closet and the book is on the desk,” “The small green frog sat on a log and caught a lot of flies”).

On each trial, the textual phrase and one of four specific emotions were projected simultaneously on a screen located approximately four meters in front of the singers. The singers were asked to express one of four emotions (irritation, happiness, sadness and neutral/no emotion). Then a recording of the melody was played, followed by four metronome beats that signaled to the singers to begin singing the scripted phrase. Each motion capture recording was initiated when the experimental melody ended and the first metronome beat began. The motion capture recording ended four to five beats after the singing ceased. In total, there were 112 recordings (7 musicians × 4 emotions × 4 phrases).

#### Point-light stimulus creation

All motion capture stimuli were gap-filled and cleaned to ensure that marker trajectories appeared natural. The shoulder marker was removed from the data set. The spatial trajectories of the remaining 28 markers were smoothed to reduce measurement noise. Smoother trajectories were estimated from the original data using Functional Data Analysis (FDA; Ramsay and Silverman, 2005). This analysis method converts the discrete measurements into continuous functions based on b-splines with a roughness penalty λ set to 10^−12^ applied to the second derivative (acceleration). All recordings were numerically centered by making the origin equivalent to the approximate center of head rotation (located in the neck). The six independent head motion parameters (three translational, three rotational) were estimated from three markers (nasion, right temple, left temple), which were assumed to have moved only due to rigid head motion with no or very little interference from non-rigid skin motion. The standard estimation algorithm based on Procrustes Analysis (Gower, 1975) showed small residuals confirming that the markers were largely unaffected by skin movements.

Data for the three epochs were extracted from the full recordings in the following way. First, two researchers independently determined the onset of the first sung syllable, based on acoustic inspection. In most cases, the judgments were based on the acoustic signal. In a few instances, however, the acoustic signal was missing and the onset and offset of facial singing movements had to be visually approximated and so provided the only criterion for a decision. The average difference between the raters in start times was 10 frames (=50 ms) and for end times was 33 frames (=165 ms).

For the pre-production epoch, data samples from 1.5 s before the onset of the singing were selected. For the production epoch, samples corresponding to a duration of 1.5 s centered on the midpoint of the sung phase were selected. For the post-production epoch, data samples starting with the offset of the singing and extending to 1.5 s beyond this point were selected. The marker data was turned into video clips of point-light displays without any other modifications. Each marker was represented by a black dot moving in front of a white background. A frontal perspective was chosen to reduce the three-dimensional data to the two dimensions of the video clip. The perspective coincided with the x-axis of the Vicon coordinate system and coincided with the direction of an assumed audience during the motion capture session. The movement range across all trials was determined beforehand and the display limit was set accordingly to keep the point-lights visible at all times.

To ensure that the stimulus was recognized as a face, a brief anchor stimulus was added to the beginning of every clip. It consisted of a static point-light face generated from the reference sample (before any emotion was expressed), but with gray lines inserted between selected markers so as to emphasize salient facial features (see Figure 1). Three anatomical structures were emphasized: the mouth, by connecting lip markers; the eyebrows, by connecting medial to lateral eyebrow markers; and the nose, by connecting the nasion and the nose tip marker. The final clip consisted of the following sequence: a blank (white) screen for 0.4 s; the static anchor face for 1 s; another blank screen for 0.4 s; the point-light motion stimulus (without connecting lines) for a duration of 1.5 s; and a final blank screen for 0.4 s.

The entire processing described above was accomplished through custom-written Matlab (The MathWorks) routines. To achieve the desired video frame rate of 25 fps, the motion data were down-sampled. For each data sample, a video frame was created in the form of a Matlab figure that was subsequently added to a Quicktime movie using the Matlab Quicktime toolbox written by Slaney (1999).

#### Analysis of movement data (PCA)

The motions of the singers were assessed to quantitatively examine the changes in facial motion over time. A principal components analysis (PCA) of facial movements and head movements was conducted, using stimuli from both Experiment 1 (normal movements) and Experiment 2 (exaggerated movements). Combining stimuli from the two experiments provided us with enough observations for a robust PCA with 27 variables. The movements of the musicians were first quantified by their displacement (relative to the positions of the neutral expression at the beginning of each trial), velocity and acceleration for the points associated with the lip corners, eyebrows, front-back head movement, lateral head movement, up-down head movement, and the rotational movements of pitch, roll and yaw. PCA is an appropriate analysis because many of these motion variables were highly correlated. Before the analysis was performed, the movement variables were standardized to have the same variance. Five components emerged with eigenvalues greater than 1 (which we used as cut-off criterion). The five components accounted for 82 percent of the variation in the data.

Table 1 shows the correlation between each component and the motion variable of interest. Component 1 is associated with changes in the mouth region, Component 2 is associated with head displacement and head velocity, Component 3 is most strongly associated with head movements and rotations from side to side, Component 4 is associated with head acceleration, and Component 5 is associated with eyebrow movement.

**Table 1:** **The principal component scores from the rotated component matrix**.

**Variable**	**Rotated component matrix**				
	**Component**				
	**1 Mouth**	**2 Head displacement/velocity**	**3 Side head motion**	**4 Head acceleration**	**5 Eyebrows**
Mouth corner displacement	**0.724**	0.246	0.248	0.110	0.208
Eyebrow displacement	0.130	0.319	0.153	−0.115	**0.757**
Mouth area	**0.806**	0.201	0.184	0.113	−0.003
Mouth corner velocity	**0.914**	0.140	0.171	0.111	0.198
Eyebrow velocity	0.282	0.149	0.223	0.138	**0.877**
Mouth area velocity	**0.928**	0.154	0.165	0.104	0.081
Mouth corner acceleration	**0.867**	0.044	0.133	0.201	0.275
Eyebrow acceleration	0.227	−0.037	0.202	0.321	**0.793**
Mouth area acceleration	**0.912**	0.113	0.140	0.135	0.135
Head front back displacement	0.119	**0.699**	0.381	−0.141	0.133
Head lateral displacement	−0.017	0.436	0.552	0.311	0.013
Head up down displacement	0.088	**0.808**	0.220	0.305	0.025
Head front back velocity	0.262	**0.638**	0.569	0.080	0.154
Head lateral velocity	0.148	0.399	**0.690**	0.351	0.058
Head up down velocity	0.152	**0.652**	0.321	0.598	**−**0.016
Head front back acceleration	0.431	0.327	0.545	0.410	0.272
Head lateral acceleration	0.370	0.234	**0.720**	0.366	0.220
Head up down acceleration	0.257	0.358	0.330	**0.749**	0.066
Head rotation roll displacement	0.197	**0.709**	0.303	0.136	0.126
Head rotation pitch displacement	0.193	**0.774**	0.108	0.234	0.188
Head rotation yaw displacement	0.143	0.427	**0.740**	0.108	0.203
Head rotation roll velocity	0.233	**0.664**	0.429	0.382	0.132
Head rotation pitch velocity	0.289	**0.634**	0.242	0.505	0.211
Head rotation yaw velocity	0.245	0.298	**0.841**	0.103	0.206
Head rotation roll acceleration	0.337	0.443	0.519	0.487	0.219
Head rotation pitch acceleration	0.431	0.269	0.237	**0.674**	0.340
Head rotation yaw acceleration	0.361	0.150	**0.810**	0.114	0.251

*Large correlations >0.6 are in bold*.

#### Differences between epochs

**Figure 3:**
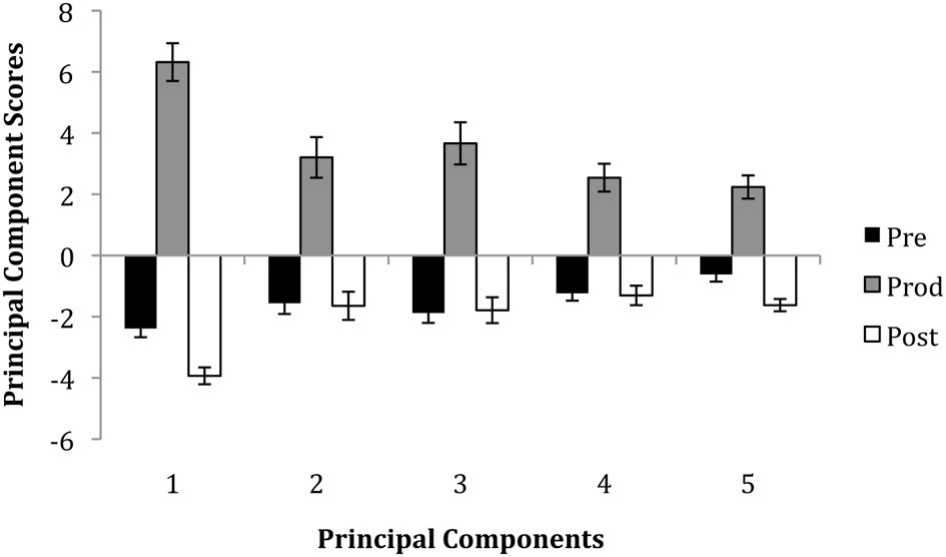
**The average principal component scores for each epoch**. Error bars represent standard errors.

The average component scores for each epoch are shown in Figure 3. The graph shows that, not surprisingly, there were higher scores in the production epoch for every component as compared to the pre- and post-production epochs. This reflects the larger movements that were used by singers during singing. The figure also shows that there was less movement in the post-production epoch than the pre-production epoch—particularly for the 1st and 5th components, which are associated with mouth and eyebrow movements respectively.

#### Individual differences between singers

**Figure 4:**
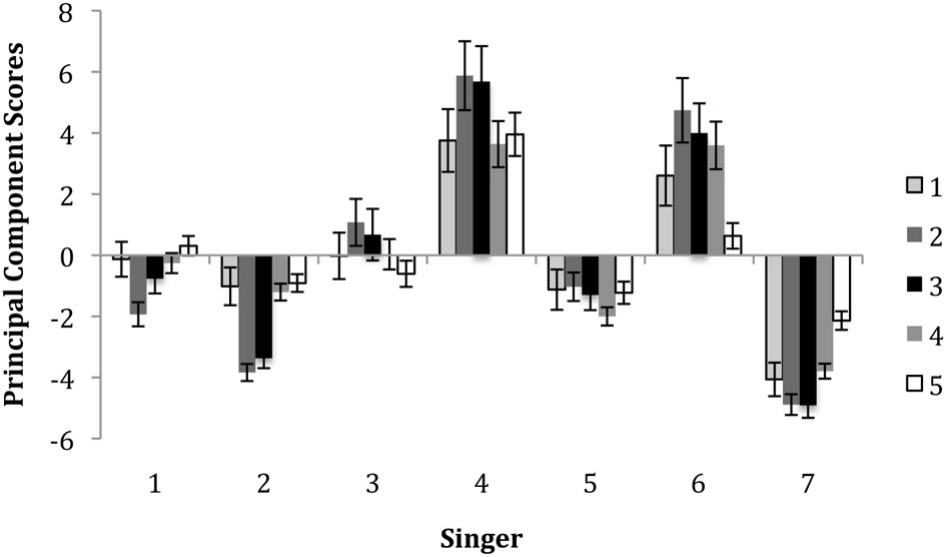
**The average principal component scores for each singer**. Error bars represent standard errors.

An analysis of differences in the use of movements by singers, as reflected by component scores, was performed. A multivariate analysis of variance with singer (7) as the independent variable and the 5 components as the dependent variables showed that singers may have used somewhat different strategies to encode their emotional intentions. There were significant differences between singers in each of the five components, all *F*s > 11.46, *p*'s < 0.001. Figure 4 illustrates the average principal component (PC) values for each singer and indicates individual differences in facial movement across features. The averaging over the five principal components gives an indication of overall movement across features. Generally, Singers 4 and 6 used more extensive movements than other musicians. Singer 4 showed prominent eyebrow movement (Component 5), mouth movement (Component 1) and head movement (Components 2–4) when compared to other singers. In contrast, Singer 6 used more extensive head movement (Components 2–4) than the other singers. Singer 7 used smaller facial and head movement than the other singers, with the exception of Singer 2, who used very little head movement. The analysis of the motion data revealed that there were several aspects of motion associated with the expression of emotion by the singers. Singers used facial expressions (mouth and eyebrow movement) and head movement to express emotion. Individual singers also varied in their overall use of motion and in the specific movements that they employed.

### Emotional decoding

#### Participants

Sixteen members of the Macquarie University community including researchers, graduate students and post-doctoral fellows (11 females and 5 males) participated in Experiments 1–3, during which they provided ratings of 336 stimuli. There were 1344 conditions (7 musicians × 4 emotions × 3 epochs × 2 exaggeration × 2 scrambled × 4 phrases) but each participant only rated one phrase. The average age of the participants was 37.75 (*SD* = 15.16; range = 21–62) years. Although each experiment was not independent (the same viewers participated), the analyses between variables are reported separately to allow for ease of interpretation.

#### Materials and procedure

The point-light stimuli were presented on an Apple Macintosh iMac12.2 with an integrated 27 inch monitor that had 2560 × 1440 pixel resolution and was situated in a quiet room. The participants were seated with their face approximately 60 cm away from the monitor, such that the stimulus area subtended a visual angle of roughly 11 degrees. Stimuli were presented in six blocks, with different epochs (pre-production, production, post-production) and scrambling mode (see Experiment 3) presented in separate blocks. To reduce the length of the experiment, the 16 participants were randomly and independently assigned in sets of four to stimuli containing only one of the four text phrases. The exaggerated stimuli (Experiment 2) were presented in the same blocks as the normal stimuli, as these stimuli met the expectations for biological motion.

Custom-written software was programmed in Python and a web-based framework was used to show the movie clips and obtain the ratings from the participants. For each trial, there were four slider scales labeled “Happiness,” “Irritation,” “Sadness,” and “Neutral” ranging from 1 (“*not at all*”) and 7 (“*very much*”). The four sliders appeared horizontally stacked underneath the area where the movie was displayed. The stack order was randomized across blocks. The participants were instructed to first watch the movie and then rate the perceived strength of the emotion expressed by the point-light face by moving the sliders with the computer mouse to a position between 1 and 7. In the pre-production epoch, participants were instructed to rate the extent to which the singer moved toward conveying a particular emotion (i.e., from neutral to some emotion). In the production epoch, participants were instructed to rate the extent to which the singer conveyed a particular emotion. In the post-production epoch, participants were instructed to rate the extent to which the singer moved away from conveying a particular emotion (i.e., from an emotion toward neutral). The participants were able to use more than one scale to indicate a mixture of perceived emotions and were made aware of this option. Once they were satisfied with their ratings they continued to the next trial. There was no audio associated with any of the stimuli.

### Results

Three hundred and thirty-six conditions were analyzed in a mixed-design analysis (4 emotions × 4 phrases × 7 singers × 3 epochs), with 84 trials rated per viewer (one phrase). The exaggerated and scrambled conditions were assessed in Experiments 2 and 3. To assess the accuracy of emotional decoding, the emotion ratings were first converted to correct/incorrect responses. The response was considered “correct” if the highest rating of the four emotional ratings matched the emotion communicated and “incorrect” otherwise. For example, if the intended emotion was assigned a rating of “2” and the remaining options were assigned ratings of “1,” the intended emotion was still considered correct as this option had the highest rating relative to the incorrect options. Cases in which participants rated two emotions equally high (one matching the intended emotion and the other not matching the intended emotion) were coded as incorrect (*n* = 48).

#### Correct responses by epoch, singer and emotion

**Figure 5:**
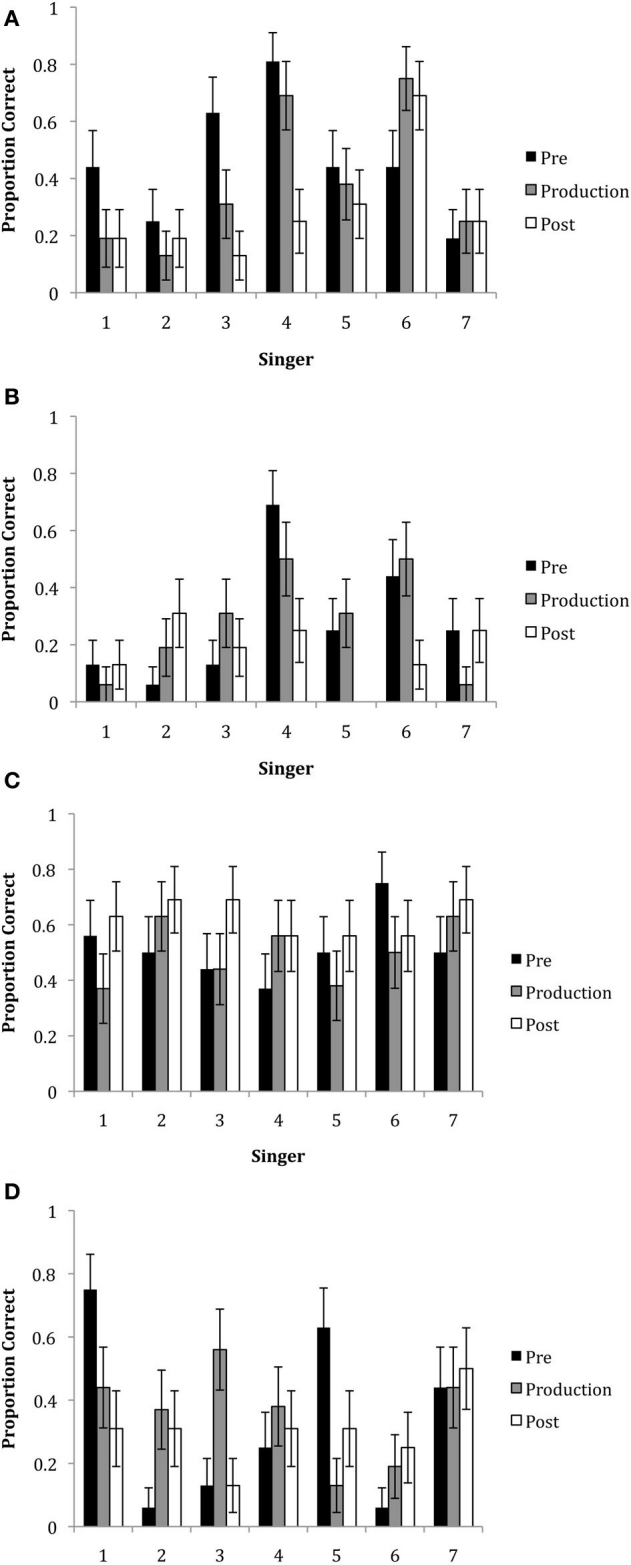
**The proportion of correct responses for the emotions of **(A)** happiness, **(B)** irritation, **(C)** neutral and **(D)** sadness for each of the seven singers in each of the three epochs**. Note that missing bars indicate that no participant accurately decoded the emotional intention.

In all three experiments, decoding accuracy did not differ between phrases, therefore these conditions were combined. A GLM analysis including the factors of epoch, singer, emotion and all interactions was performed. Figures 5A–D show the mean ratings by emotion, epoch, and singer. Overall, the mean correct responses (*M* = 37.43; *SE* = 7.26) indicated that emotions were decoded at above chance levels. There was a main effect of emotion, *F*_(3, 1245)_ = 27.44, *p* < 0.001. This reflected the finding that neutral and happiness were decoded more accurately than irritation and sadness. There was also a main effect of singer, *F*_(6, 1245)_ = 3.20, *p* = 0.004. Generally, this showed that Singer 4 was most able to communicate expressively across emotions as compared to the other singers. There was also a significant emotion x singer interaction, *F*_(18, 1245)_ = 3.903, *p* < 0.001, which showed that some singers were better at communicating particular emotions than other singers. For example, happiness was best decoded when expressed by Singers 4 and 6, irritation was best decoded when expressed by Singer 4, and sadness was best decoded when expressed by Singers 1 and 7.

Although there was no significant main effect of epoch, *F*_(2, 1245)_ = 1.29, *p* = 0.279, there were significant interactions of epoch with other variables: between epoch × emotion, *F*_(6, 1245)_ = 2.520, *p* = 0.020; and epoch x singer, *F*_(12, 1245)_ = 2.208, *p* = 0.010. The 2-way interaction for epoch x emotion showed that happiness was generally better decoded in the pre-production epoch (*M =* 45.53, *SD* = 50.02) than the post-production epoch (*M* = 28.57, *SD* = 45.37), *t*_(15)_ =2.83, *p* < 0.014. The epoch by singer interaction showed that overall, Singer 1 was best able to express emotion in the pre-production epoch as compared to the production and post-production epochs and Singer 4 was marginally better at communicating emotions in both the pre-production and production epochs as compared to the post-production epochs.

Finally, there was a 3-way interaction with epoch x singer x emotion, *F*_(36, 1245)_ = 1.781, *p* = 0.003. Tests of simple effects with Bonferroni correction showed that there were no significant differences across epochs for Singer 2, Singer 6 and Singer 7. Singer 3 and Singer 4 were better able to express happiness in the pre-production epoch as compared to the production epoch, *t*_(15)_ = 3.16, *p* < 0.005 and *t*_(15)_ = 3.65, *p* < 0.001, respectively. Singer 4 was better able to communicate happiness in the production epoch, *t*_(15)_ = 2.76, *p* < 0.017, as compared to the post-production epoch. Singer 4 also communicated irritation better in the pre-production epoch than the post-production epoch, *t*_(15)_ = 2.76, *p* < 0.017. Singer 1 was best able to communicate sadness in the pre-production epoch as compared to the post-production epoch, *t*_(15)_ = 2.76, *p* < 0.017, while Singer 5 was better able to express sadness in the pre-production epoch as compared to the production epoch, *t*_(15)_ = 3.16, *p* < 0.005. Singer 3 was best able to express sadness in the production epoch as compared to the pre- and post-production epochs, *t*_(15)_ = 2.76, *p* < 0.017.

### Discussion

The findings of Experiment 1 showed that expressions of happiness and neutral were more likely to be perceived by viewers from point-light displays of singers' facial features compared to expressions of irritation and sadness. Although anger and sadness may be communicated in full-video (Dahl and Friberg, 2007), previous work using PLDs has shown that the emotions of anger and sadness may not be as well communicated as happiness in PLDs (Bassili, 1979). The results also showed that emotional decoding was dependent on the singer and epoch. Perceivers were better able to decode emotions in the pre-production and production epochs, as compared to the post-production epoch. Generally, happiness was more clearly decoded in the pre-production epoch than the production epoch. This is consistent with previous findings, suggesting that happiness is a difficult emotion to convey during singing because facial areas signaling happiness are being recruited (Scotto di Carlo and Guaitella, 2004). For some singers (4, 5, 6), perceivers decoded irritation better in the pre-production epoch as compared to the post-production epoch. Similarly, perceivers were better able to decode sadness when communicated by Singer 1 and Singer 5 in the pre-production epoch as compared to the post-production and production epochs respectively. Cues to anger and sadness might be found higher in the face in the form of a frowning motion or raised eyebrows (Bassili, 1979). Due to the restrictions involved in singing, singers conveyed some of the cues just before singing, while other cues, such as eyebrow movements and head movements could be used during singing.

We did not find a strong effect of post-production lingering, at least with regard to emotional decoding. We might infer that from the perspective of the viewing participants, once singers had completed singing, there was not much available evidence for participants to determine the emotion. These findings at first seem to contrast with those of Livingstone et al. (2009), who found that both with motion capture and with EMG, musicians “lingered” or maintained the displacement from the production phase into the post-production phase. However, one important difference between these studies is that Livingstone et al. focused on the production of emotional singing and did not examine emotional decoding. It is possible that musicians in our study did emotionally “linger” or prepare but this may not have been sufficient for perceiving participants to determine the emotional intention in PLDs.

## Experiment 2

The findings of Experiment 1 showed that happiness and neutral were more likely to be decoded by viewers than irritation and sadness. Importantly, several singers expressed the emotion of happiness through facial expressions even before they began singing. Given the modest levels with which the emotional intentions were decoded, Experiment 2 was designed to evaluate whether emotional cues were present but were too subtle for perceivers, based on facial (visual) cues. That is, singers may have encoded the emotion in facial expressions but such movements may not have been sufficiently clear to perceivers, especially when presented as PLDs.

To evaluate this possibility, the PLDs in Experiment 2 were manipulated so that facial movements were exaggerated twofold. This manipulation was performed to assess whether the relevant emotional information was present in facial movements but not adequately detected by perceivers. We expected that exaggerated movements would be more accurately decoded than non-exaggerated movements, because exaggerated movements should convey greater emotional intensity (Pollick et al., 2003). Indeed, a comparison of performance movements for deadpan and expressive performances revealed that the movements used in expressive performances are similar to, but larger than the movements used in deadpan performances (Davidson, 1994; Wanderley et al., 2005). That is, exaggerated movements may enhance the expressiveness of facial movements, leading to increased decoding accuracy. However, exaggerating the temporal and dynamic characteristics of the motion may actually lead to reduced decoding accuracy for some emotions that may rely on slower movements (e.g., sadness; Kamachi et al., 2001; Sato and Yoshikawa, 2004; Recio et al., 2013).

### Methods

#### Participants

The participants were the same 16 individuals from Experiment 1. Technically, Experiments 2 and 3 might be considered separate conditions rather than experiments; however, these conditions were separated to make interpretation clearer. A caveat of this approach is that participants were exposed to all the stimuli, which might have biased responses in various conditions. That is, participants' ratings may have been influenced by stimuli to which they had previously been exposed.

#### Point-light creation

We created exaggerated stimuli by multiplying the original head motion parameters and face motion trajectories by a factor of two. This doubled the distance of each marker trajectory while keeping the time constant. However, the velocity of the marker movement was also increased. This level of exaggeration was selected with the aim of maximizing the impact of the resultant movements without appearing to be biologically impossible. The reference positions for each trajectory were subtracted from the entire trajectory before being exaggerated. As each trial always commenced with a neutral facial expression (which was used as an anchor before participants saw the dynamic PLDs), these expressions determined the reference positions. In this analysis, only the exaggerated stimuli were considered.

### Results

#### Emotional decoding

**Figure 6:**
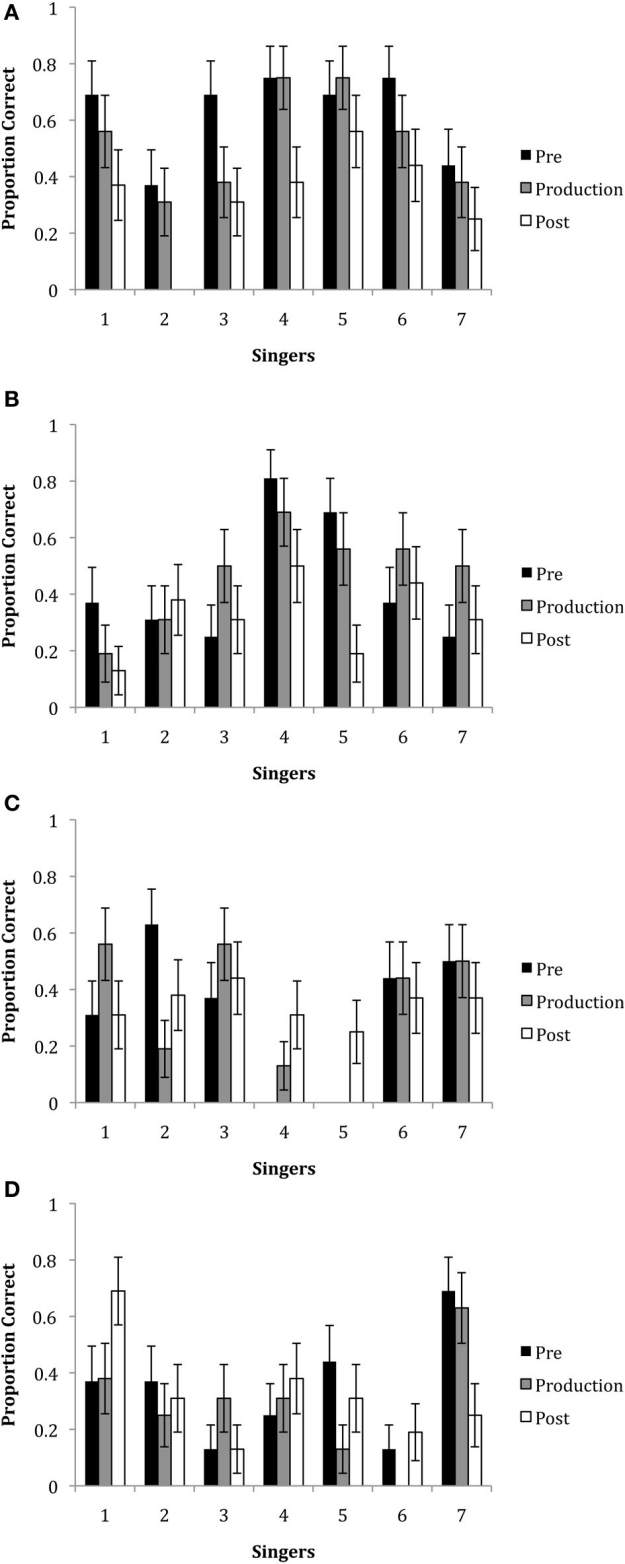
**The proportion of correct responses for the emotions of **(A)** happiness, **(B)** irritation, **(C)** neutral and **(D)** sadness for each of the seven singers in each of the three epochs using the exaggerated stimuli**. Note that missing bars indicate that no participant accurately decoded the emotional intention.

Three hundred and thirty-six conditions were analyzed (4 emotions × 4 phrases × 7 singers × 3 epochs); each viewer rated 84 trials (one phrase) for the exaggerated movement stimuli. As before, trials were considered incorrect when participants rated two emotions equally high (one matching the intended emotion and the other not matching the intended emotion; *n* = 55). Overall, the mean correct responses (*M* = 38.91; *SE* = 7.63) indicated emotions were decoded above chance levels. A GLM analysis including the factors of epoch, singer, emotion and all interactions was performed on the percent correct values. Figures 6A–D show the mean responses by epoch, singer, and emotion. The results showed that there was a significant main effect of epoch, *F*_(2, 1245)_ = 6.172, *p* = 0.002. This finding showed that emotional decoding was better in the pre-production and production epochs than in the post-production epoch. There was also a main effect of emotion, *F*_(3, 1245)_ = 11.08, *p* < 0.001. Overall, happiness and irritation were better decoded than neutral and sadness. There was no significant effect of singer, *F*_(6, 1245)_ = 1.559, *p* = 0.156.

There were significant 2-way interactions between epoch x emotion, *F*_(6, 1245)_ = 3.520, *p* = 0.002; emotion × singer, *F*_(18, 1245)_ = 6.718, *p* < 0.001; but not epoch × singer, *F*_(12, 1245)_ = 0.742, *p* = 0.711. The two-way interactions revealed that again, happiness was better decoded in the pre-production, *t*_(15)_ = 4.91, *p* < 0.001, and production epochs, *t*_(15)_ = 3.26, *p* = 0.003, as compared to the post-production epoch. Irritation was better decoded in the production epoch as compared to the post-production epoch, *t*_(15)_ = 2.53, *p* = 0.033. The singer by emotion interaction revealed that with the exception of Singers 2 and 7, most were able to communicate happiness. Singer 4 was best able to communicate irritation. Singers 1 and 7 were best able to communicate sadness.

There was also a significant 3-way interaction with epoch × singer × emotion, *F*_(36, 1245)_ = 1.597, *p* = 0.014. Tests of simple effects with Bonferroni correction showed that there were no significant differences across epochs for Singer 1 and Singer 6. The findings showed that Singer 4 was marginally better at communicating happiness in the pre-production and production epochs as compared to the post-production epoch, *t*_(15)_ = 2.37, *p* = 0.053. Singer 2 was marginally better at communicating happiness in the pre-production epoch than the production epoch and Singer 3 was also marginally better at communicating happiness in the pre-production epoch as compared to the post-production epoch, *t*'s_(15)_ = 2.37, *p* = 0.053. Singer 4 was better able to communicate irritation in the pre-production, *t*_(15)_ = 3.16, *p* =0.005, and the production epochs, *t*_(15)_ = 2.37, *p* = 0.053, as compared to the post-production epoch. Singer 7 was better able to communicate sadness in the pre-production, *t*_(15)_ = 2.76, *p* = 0.017, and production epochs, *t*_(15)_ = 2.37, *p* = 0.053 as compared to the post-production epochs.

#### Comparison of viewers' emotion ratings with PC movement analysis

Each of the principal components was used as a predictor of viewers' emotion ratings in multiple regression analyses. This analysis assessed the association between the singers' facial motion cues and the viewers' decoding of emotion. Presumably, emotional judgments should be associated with specific cues signaling emotion. Table 2 shows the results of multiple regression analyses that predicted viewers' ratings in each emotion/epoch condition from the five principal component values of the facial movements. In the production epoch of each emotion, Component 1, which was associated with singers' mouth movements, predicted viewers' ratings of emotion. Expressions of happiness were generally associated with increased mouth movements and head displacement. Irritation was associated with increased mouth and eyebrow movements (Components 1 and 5) and this was particularly significant in the pre- and post-production epochs. Neutral expressions were generally associated with reduced movements in most of components. Finally, sadness was associated with some eyebrow movements, likely an upward movement of the inner eyebrow, head displacement and head rotation. An examination of the PLDs showed that head displacement reflected a downward motion of the head accompanied by a slight rotation.

**Table 2:** **The coefficient of determination, *F*-value and regression coefficients associated with the regression analyses with the components as predictors of emotion in each of the three epochs**.

**Emotion**	**Epoch**	***R^2^***	***F***	**Components**				
				**1 Mouth**	**2 Head displacement/velocity**	**3 Side head motion**	**4 Head acceleration**	**5 Eyebrows**
Happiness	Pre	0.22	11.99	0.15^*^	0.13^*^	−0.07	−0.09	0.01
	Production	0.31	19.96	0.08^**^	0.09^*^	−0.04	−0.09	0.03
	Post	0.20	10.59	0.17^**^	0.03	−0.01	−0.04	−0.10^*^
Irritation	Pre	0.16	8.12	−0.08	0.08	0.03	−0.13^*^	0.21^***^
	Production	0.24	13.87	0.09^***^	0.00	0.00	−0.04	0.01
	Post	0.14	7.33	0.03	0.02	−0.04	0.01	0.08^*^
Neutral	Pre	0.34	22.89	−0.11^*^	−0.18^**^	0.06	0.30^***^	−0.18^**^
	Production	0.34	22.45	−0.12^***^	−0.12^**^	0.06	0.17^**^	−0.04
	Post	0.31	19.78	−0.16^*^	−0.12^**^	0.05	0.24^***^	−0.18^**^
Sadness	Pre	0.10	4.54	−0.02	0.00	−0.03	−0.09	0.07
	Production	0.21	11.45	−0.05^*^	0.04	0.00	−0.04	−0.01
	Post	0.10	4.61	−0.07	0.12^**^	−0.12^*^	−0.09	0.21^***^

* p < 0.05,

** p < 0.01,

*** *p < 0.001*.

Overall, perceivers were sensitive to specific cues in singers' facial movements for decoding emotion. The high decoding of happiness in the pre-production epoch may have occurred because there was considerable movement of the mouth corner in the pre-production epoch when compared to the production epoch. Perceivers associated eyebrow movements with the expression of irritation. The expression of neutral was associated with less movement overall. Sadness was associated with eyebrow movement and head displacement.

### Discussion

The findings of Experiment 2 suggest that emotional information can be conveyed in the PLDs of exaggerated face and head movements. The overall accuracy of emotional decoding appeared to be similar between Experiments 1 and 2. In Experiment 2, the emotions of happiness and irritation were particularly well decoded. The exaggerated movements may mainly disambiguate high-arousal emotional intentions (i.e., happiness and irritation) from low-arousal emotional intentions (i.e., neutral and sadness). Consistent with this interpretation, the high arousal emotions of irritation and happiness were generally well identified. In contrast, sadness was not well decoded.

There appeared to be differences in the decoding of exaggerated facial movements depending on the epoch. In particular, perceivers were able to decode happiness in several singers before they began singing. Perceivers were able to decode emotion from specific singers when they expressed irritation (Singer 4) and sadness (Singer 7) in the pre-production and production epochs as compared to the post-production epoch. These findings suggest that the expressive intentions of some singers were perceptible outside of the timeframe of singing when the movements were exaggerated.

## Experiment 3

Experiment 2 demonstrated that facial movements associated with high-arousal emotions (happiness and irritation) were decoded accurately by viewers when they were algorithmically exaggerated in range of motion. Experiment 3 addressed whether the extent of motion, rather than the specific motion configuration associated with singing, communicates important emotional information. Although unlikely, it is possible that the decoding of emotion might have been based solely on the magnitude of motion information rather than the particular expressive movements. That is, it is possible that the coherence or configuration of the marker information may not have been necessary to decode emotion.

Experiment 3 presented “scrambled” motion configurations of the same facial movements used in previous experiments. The direction of marker movement commenced from a randomly determined position. The marker trajectory could be in any 360 degree direction. That is, the marker appeared in the neutral starting position, and then moved with the same acceleration, velocity, and distance as in the original stimulus but in a randomly determined trajectory that was independent of other markers. The manipulation was introduced to all stimuli from Experiments 1 and 2, and included both the original and exaggerated stimuli. It was predicted that perceivers would be sensitive to the overall amount of displacement, velocity, and acceleration of point-light movements, which may contain information about the level of arousal of the intended emotion. However, such a manipulation removes configural information (e.g., features associated with a smile or a frown), and motion coherence, which may be important for differentiating emotions that are similar to each other in their level of arousal. Previous work has demonstrated that the relative positions and timing of markers in PLDs are needed for accurate perception (e.g., Bertenthal and Pinto, 1994). In the absence of configuration information, we expected emotions to be less accurately decoded. However, because randomized movements should reflect overall arousal levels, we reasoned that viewers might be most accurate at decoding low arousal emotions such as sadness and neutral when the scrambled stimuli were not exaggerated, and most accurate at decoding high arousal emotions when the scrambled stimuli were exaggerated.

### Methods

Scrambled stimuli were created by randomly changing the orientation of movement as it appeared in the two-dimensional image plane. This occurred for the trajectories of all markers without respect to the rigidity or non-rigidity of the movements. The location of the first sample of each trajectory of each trial was used as the center of rotation and determined the randomly selected direction that the marker would travel. The first frame of each trial showed an unscrambled face, while in the following frames, the face tended to immediately disintegrate or jitter, due to the markers moving in random directions with the same amount of displacement, speed, and acceleration of the original trajectories. All other values associated with the movement were retained, including the maximum, minimum and standard deviations associated with individual marker movement. In many cases, the stimulus no longer resembled a moving face and head because individual marker movements no longer conformed to the configuration found in a face. The scrambling was applied to both types of trials—the original motion and the exaggerated motion.

### Results

Six hundred and seventy-two conditions were considered in this analysis (4 emotions × 4 phrases × 7 singers × 3 epochs × 2 exaggeration), with each viewer rating 168 trials (one phrase). There were more conditions in this analysis than in Experiments 1 or 2 because participants rated both the original stimuli and the exaggerated stimuli. As before, trials on which participants rated two emotions equally high (one matching the intended emotion and the other not matching the intended emotion) were considered incorrect (*n* = 145). Table 3 displays the mean percent correct for each condition. Overall, the mean correct responses (*M* = 28.23, *SE* = 6.37) indicated that emotions were decoded at chance levels. A GLM analysis including the factors of epoch, emotion, and exaggeration with all interactions was performed. The results showed that there was a significant main effect of emotion, *F*_(2, 2664)_ = 16.12, *p* = 0.001, such that stimuli expressing happiness and neutral were better decoded than stimuli expressing neutral and sadness. There was a main effect of epoch, *F*_(2, 2664)_ = 4.16, *p* = 0.02, showing that stimuli in the production epoch were better decoded than the stimuli in the post-production epoch. There was no main effect of exaggerated stimuli, *F*_(1, 2664)_ = 0.017, *p* = 0.89, suggesting that exaggeration alone did not suggest any one particular emotion. However, there was a significant interaction between emotion and exaggeration, *F*_(3, 2664)_ = 24.91, *p* = 0.001. When happiness and irritation were exaggerated, these emotions were better decoded than the emotions of neutral and sadness. There was no significant interaction between epoch and emotion, *F*_(6, 2664)_ = 0.68, *p* = 0.66, and between epoch and exaggeration, *F*_(2, 2664)_ = 0.62, *p* = 0.54. The 3-way interaction between epoch, emotion and exaggerated was marginally significant, *F*_(36, 2664)_ = 1.95, *p* = 0.07. This reflected the trend that exaggerated stimuli expressing happiness were better decoded in the production epoch as compared to the post-production epoch.

**Table 3:** **The average accuracy ratings for each emotion and epoch for the original and exaggerated scrambled stimuli**.

**Emotion**	**Epoch**					
	**Pre**		**Production**		**Post**	
	**Spatial type**		**Spatial type**		**Spatial type**	
	**Original**	**Exaggerated**	**Original**	**Exaggerated**	**Original**	**Exaggerated**
Happiness	25.89 (44.00)	**39.28** (49.01)	25.89 (44.00)	**46.42** (50.09)	26.78 (44.48)	28.57 (45.37)
Irritation	25.00 (43.49)	25.89 (44.00)	20.53 (40.57)	**38.39** (48.85)	11.61 (32.18)	30.35 (46.18)
Neutral	**41.07** (49.42)	23.21 (49.41)	**52.68** (50.15)	27.67 (44.94)	**49.99** (50.22)	21.42 (41.22)
Sadness	22.32 (41.83)	20.53 (40.57)	23.21 (42.41)	19.64 (39.91)	18.75 (39.21)	19.64 (39.91)

*Standard deviations are in parentheses. Values in bold indicate above chance decoding*.

### Discussion

The results of Experiment 3 show that when the relative global relationships between point-lights are removed and only motion information is maintained, perceivers had difficulty decoding the emotional expression. This finding suggests that range, velocity, and acceleration of motion (which were the same for scrambled and biological movements) were not the sole determinants of viewers' emotional ratings of singers' facial movements. Instead, specific configural information and motion coherence about facial movements, which was lost in the scrambled versions, guided viewers to more accurate ratings of the biological facial movements. The manipulation also helped to clarify some of the strategies used by participants as they attempted to decode emotional intentions. In the original condition, participants tended to assign high ratings of neutral expression, possibly because they found the movements to be ambiguous or uninterpretable. However, when movement was exaggerated, viewers were likely to perceive the emotions of happiness and irritation in the production epoch. This finding suggests that the arousal level of an emotion can be conveyed in the absence of relative global information, but only when the magnitude of the motion information is obvious and over a longer duration (as in the production epoch). Despite the presentation of the experimental conditions being counterbalanced, one possibility is that because the same observers participated in the experiments they were aware that greater motion was associated with happiness and irritation. We do not believe that this is likely due to the higher accuracy for happiness and irritation in only the exaggerated condition. If participants were primed, then we would have expected higher decoding accuracy in the original condition for these emotions.

## General discussion

In three experiments, we examined the communication of singers' emotions based on facial movements before, during, and immediately after singing. The findings suggest that singers use facial expressions and head movements in ways that correlate with the intended emotion. Perceivers, in turn, interpret the movements used by singers and can decode intended emotions. However, accurate decoding depends on the intended emotion, the epoch, and the singer. The emotional connotations of certain movements can be clarified when the recorded movements are exaggerated, especially for high-arousal emotions. Exaggerating the movements associated with a low-arousal emotion, however, can suggest a high-arousal emotion, leading to lower decoding accuracy. Removal of configural cues (through randomizing movement) leads to low decoding rates, suggesting the importance of configural cues; however, overall arousal information may be preserved in randomized movement. Finally, significant individual differences were observed: singers differed from each other in their use of facial and head movements in pre-production, production, and post-production epochs, leading to differential decoding rates for each singer at different temporal epochs. These findings will be discussed in turn.

First, our results corroborate a growing body of evidence that singers use facial expressions and head movements in ways that correlate with expressive intentions, including emotional intentions (Davidson, 1993, 1994, 1995; Thompson et al., 2005, 2008, 2010; Livingstone et al., 2009). The findings from Experiments 1 and 2 showed that perceivers were sensitive to eyebrow and lip movements. Inspection of the videos showed that singers frowned or raised their eyebrows to signal irritation and sadness respectively, and smiled to signal happiness. For all singers, the amount of displacement, velocity, and acceleration varied as a function of the intended emotion. Happiness was associated with higher values on all these motion variables whereas sadness was associated with lower values. These commonalities between individual musicians allow perceivers to use consistent strategies when decoding emotional intentions. This finding is consistent with Brunswik's Lens model in that some cues must be common amongst all senders for receivers to be able to decode the senders' intentions.

Head movements were also used to express emotion although perceiver's judgments seemed not to significantly associate head motion with any one particular emotion. Head movements make performances more natural, expressive, and signal cycles of tension and relaxation (Wanderley et al., 2005; Busso et al., 2007; Castellano et al., 2008). Moreover head movements alone have been found to communicate emotion (Dahl and Friberg, 2007).

Second, we observed that perceivers could interpret emotional information from face and head movements not only during singing, but prior to the onset of singers' vocalization. In Experiments 1 and 2, viewers were able to decode happiness before singing commenced. This effect, however, did not reliably extend to the other emotional intentions and was not evident in the post-production epoch.

Currently, more research is needed to better understand the phenomenon of emotional preparation and lingering. Our findings are consistent with Livingstone et al. (2009), in that across epochs, musicians used movements as a form of expression. The current study found that perceivers could not meaningfully use the information in the post-production epoch to decode emotions. There are a few possibilities for this outcome. The first is that singers used movements in the post-production epoch but not to the same extent as in the pre-production and production epochs, particularly mouth and eyebrow movements. A second is that moving “away” from an emotion is unnatural for perceivers to decode. Some evidence for this possibility comes from the finding that participants are poorer at decoding emotion from full-video sequences that are shown backwards as compared to forwards (Cunningham and Wallraven, 2009; Experiment 4). Firm comparisons between Livingstone et al. (2009) and our work are difficult to make due to fundamental differences in methods (i.e., the use of point-light displays vs. videos; assessment of emotional decoding vs. emotional mimicry in viewers).

Third, the results showed that exaggerating movements sometimes assisted in emotional decoding although the manipulation may have distorted the emotional expression. In Experiment 2, the emotions of happiness and irritation were well decoded as compared to neutral and sadness. The high decoding of these emotions may be attributable to high arousal emotions being associated with greater displacement, velocity, and acceleration than low arousal emotions. These findings are also consistent with past work demonstrating that exaggerated movements lead to higher ratings of emotional intensity (Pollick et al., 2003). The data suggest that exaggeration of the motion did not benefit the decoding of sadness. When movements expressing sadness were exaggerated in Experiment 2, the movements were often no longer consistent with the expression of this emotion in some singers. Accurate decoding of sadness may rely on information that is consistent with the expected motion information. For example, the expression of sadness unfolds more slowly than other emotions and when its development speeds up, it is no longer perceived as natural (Kamachi et al., 2001; Sato and Yoshikawa, 2004). In our stimuli, sadness was only well decoded for musicians who showed minimal movement and for whom the exaggeration manipulation would not have affected as strongly.

Fourth, the poor decoding accuracy observed in Experiment 3 confirms that configural cues and information associated with the direction of movement for individual markers are important for accurate decoding. Motion data alone (displacement/distance travelled, velocity and acceleration) may help viewers to differentiate emotional vs. non-emotional stimuli, but these data do not appear to provide sufficient information for accurate decoding. Our results seemed to show that greater degrees of movement were associated with higher arousal—analogous to findings for inverted point-light biological motion (Dittrich et al., 1996; Clarke et al., 2005). Scrambling movements may have other unintended effects though. It is possible that without the configural information and coherence between individual marker movements, participants were not able to effectively make use of motion information. That is, while the distance travelled, velocity and acceleration for each marker was the same in the scrambled conditions as in the biological conditions, participants may be even more disadvantaged by motion not being biologically possible or coherent. The interaction between motion and form information is still an area of debate. Thirkettle et al. (2009), using PLDs, found that both form and motion information were important to discrimination of human motion. Future work comparing various control conditions may reveal whether or not scrambling motion is an effective control condition or has other unintended effects (Hiris, 2007; Thirkettle et al., 2009).

Fifth, there were individual differences in emotional expression. For example, Singers 4 and 6 were generally able to express irritation and happiness more clearly than other musicians. Yet interestingly, viewers did not seem to be able to decode the emotion of sadness when expressed by these singers. This may be due to their use of larger movements. Singer 4 also exhibited highly expressive eyebrows and control over corrugator supercilli and procerus muscles—those involved in frowning (Ekman and Friesen, 1976). In contrast, perceivers tended to identify sadness more clearly when communicated by Singers 1, 3, 5, and 7. However, this depended on the epoch. One common factor was that these singers used reduced movements and showed specific facial or head cues, such as raised eyebrows. These findings show individual differences in emotional decoding. The strategies adopted by some individuals may have enhanced their ability to express some emotions at the expense of others. With many signals present, the cues were used probabilistically but perceivers may have had difficulty ignoring idiosyncratic movements when decoding emotion.

The modest decoding accuracy in the pre- and post-production epochs might be contrasted with the rich auditory and visual information musicians are able to use in performances. Point-light displays of a second and a half in duration are highly impoverished relative to full videos (even if considerable information is conveyed; Blake and Shiffrar, 2007). Decoding emotions from full-face, synthesized dynamic motions may take as long as 2.5 to 3 s for happiness and disgust, respectively (Gutiérrez-Maldonado et al., 2014). Controlled exposures to static images reveal that happiness can be decoded after 25 ms but that other emotions require more time. When free responses are measured, at least a second is required to accurately decode emotion from static images (Calvo and Lundqvist, 2008). Furthermore, comparisons of emotional decoding for PLDs are generally much lower than would be expected for full static images showing the facial expression (Bassili, 1979).

To conclude, musicians used facial and head movements to communicate emotions, and viewers were generally sensitive to these signals. There are idiosyncratic patterns in the use of these movements, and their development over time. Musicians can use pre- and post-production facial movements to supplement and surround the acoustic channel to support emotional communication. These expressions may be especially important given that movements during vocalization are heavily constrained by production. However, the influence of facial movements may vary from study to study and between individual musicians. When movements were artificially exaggerated, high arousal emotions were better expressed but low arousal emotions were more poorly expressed. Again, there were exceptions to this rule. Perceivers interpret overall movements in terms of general levels of arousal, while configural cues may provide detailed information about specific emotional intentions. The facial expressions of musicians are combined with the auditory domain to provide a rich audiovisual experience for listeners. This audiovisual expression of emotion may act to facilitate social interaction in daily life, but in music, it may highlight the emotional, expressive, and musical goals of the performer.

### Conflict of interest statement

The authors declare that the research was conducted in the absence of any commercial or financial relationships that could be construed as a potential conflict of interest.
