# Supplementary material for: Singing emotionally: a study of pre-production, production, and post-production facial expressions
Source: Front Psychol. 2014 Apr 29;5:262. doi: 10.3389/fpsyg.2014.00262 (PMC4010790; doi:10.3389/fpsyg.2014.00262)
Supplement: Supplementary file 1 [file Presentation1.PDF]

Supplemental material

Table S1

Average displacement (mm) for each singer, emotion and epoch (pre-production and production only) for the mouth corner markers.

Mouth Corner								
	Singers							Average
	1	2	3	4	5	6	7	
Pre-production								
Happiness	3.62	2.58	3.11	2.69	3.09	1.07	0.61	2.40
Irritation	1.69	1.33	1.25	2.19	2.50	1.06	0.81	1.55
Neutral	1.37	0.81	1.08	2.33	1.83	1.04	0.76	1.32
Sadness	1.92	1.46	2.05	1.69	2.37	1.06	1.07	1.66
Production Epoch								
Happiness	2.69	2.63	3.51	4.23	2.98	5.35	4.38	3.68
Irritation	3.08	3.24	2.21	2.83	3.52	3.79	2.06	2.96
Neutral	1.95	1.91	2.03	2.41	2.51	2.30	0.85	1.99
Sadness	2.65	1.92	2.54	2.16	2.32	4.52	1.19	2.47
Post-Production Epoch								
Happiness	1.35	1.06	1.41	0.83	1.40	3.53	2.53	1.73
Irritation	0.36	0.85	0.75	0.74	0.68	1.54	0.99	0.84
Neutral	0.48	0.49	0.54	0.44	0.57	0.59	0.43	0.51
Sadness	0.72	0.54	0.75	0.68	0.45	2.30	0.58	0.86

Table S2

Average displacement (mm) for each singer, emotion and epoch (pre-production and production only) for the eyebrow markers.

Eyebrow								
	Singers							Average
	1	2	3	4	5	6	7	
	Pre-production							
Happiness	0.98	0.33	1.44	2.46	1.24	0.36	0.88	0.82
Irritation	0.45	0.27	0.25	2.84	0.93	0.47	0.52	0.44
Neutral	0.25	0.28	0.29	0.97	0.70	0.30	0.27	0.81
Sadness	1.04	0.67	0.80	1.45	0.71	0.40	0.63	0.79
	Production							
Happiness	0.64	0.34	1.11	2.62	1.32	0.50	1.22	1.11
Irritation	0.24	0.58	0.44	0.79	1.03	0.85	0.85	0.68
Neutral	0.21	0.27	0.23	1.01	0.83	0.43	0.32	0.47
Sadness	0.49	0.44	0.72	0.72	0.86	0.60	0.39	0.60
	Post-Production							
Happiness	0.68	0.47	0.51	0.73	0.35	0.53	0.89	0.59
Irritation	0.26	0.41	0.26	0.46	0.20	0.77	1.60	0.57
Neutral	0.21	0.28	0.18	0.30	0.36	0.33	0.27	0.28
Sadness	0.54	0.33	0.30	0.37	0.32	1.40	0.94	0.60

Table S3

Average displacement (mm) for each singer, emotion and epoch (pre-production and production only) for front-back head movements.

Head Front-back								
	Singers							Average
	1	2	3	4	5	6	7	
	Pre-production							
Happiness	1.86	1.09	2.86	**4.23**	5.97	4.02	1.04	3.01
Irritation	3.93	1.13	2.95	**3.39**	2.94	3.19	1.04	2.65
Neutral	1.03	1.48	3.21	2.37	2.49	3.42	2.05	2.29
Sadness	**2.13**	0.74	6.7	3.71	**4.80**	2.63	1.66	3.11
	Production							
Happiness	2.32	1.24	3.90	**9.35**	2.54	**4.94**	2.67	3.85
Irritation	2.15	0.95	4.14	**5.04**	6.11	**3.30**	2.66	3.48
Neutral	1.35	0.90	1.16	2.59	6.92	1.08	1.38	2.20
Sadness	2.02	0.81	**5.40**	4.52	5.45	5.12	1.94	3.61
	Post-production							
Happiness	2.36	0.98	3.85	3.12	2.78	7.39	2.36	3.26
Irritation	7.77	0.88	4.61	6.64	5.15	1.14	1.55	3.96
Neutral	1.21	0.76	2.24	1.78	2.72	2.33	0.79	1.69
Sadness	2.11	1.28	2.34	5.67	1.39	3.86	2.03	2.67

Table S4

Average displacement (mm) for each singer, emotion and epoch (pre-production and production only) for front-back head movements.

Head Up Down								
	Singers							Average
	1	2	3	4	5	6	7	
Pre-production								
Happiness	0.57	0.36	0.68	**0.75**	0.90	2.22	0.23	0.82
Irritation	0.37	0.33	1.79	**0.79**	1.01	1.42	0.16	0.84
Neutral	0.39	0.27	0.22	0.84	0.83	1.15	0.09	0.54
Sadness	**0.27**	0.28	1.57	0.63	**1.54**	1.92	0.31	0.93
Production								
Happiness	0.60	0.35	1.30	**5.11**	1.03	**2.69**	0.54	1.66
Irritation	0.40	0.27	1.44	**2.37**	1.20	**0.85**	0.42	0.99
Neutral	0.24	0.21	0.43	1.69	0.46	0.25	0.11	0.49
Sadness	0.31	0.33	**2.82**	3.61	0.54	2.01	0.33	1.42
Post-production								
Happiness	0.59	0.44	1.15	3.24	0.80	4.02	0.62	1.55
Irritation	1.99	0.30	1.25	1.24	0.47	1.62	0.38	1.04
Neutral	0.49	0.19	0.41	0.50	0.54	0.29	0.09	0.36
Sadness	0.74	0.36	0.98	1.11	0.28	2.54	0.22	0.89

Table 2(a-c)

Principal component scores by emotion and epoch for pre-production (a), production (b), and post—production (c). Standard deviations are shown in parentheses

**Pre-production**					
	Principal Components				
	1 Mouth	2 Head displacement	3 Yaw	4 Velocity/ acceleration	5 Eyebrow
Happiness	-0.20	0.30	-0.22	0.14	0.89
	(5.32)	(6.26)	(6.05)	(4.46)	(4.23)
Irritation	-2.73	-2.16	-2.44	-1.78	-0.84
	(4.25)	(5.09)	(4.60)	(3.20)	(3.71)
Neutral	-3.48	-3.01	-2.74	-1.83	-1.55
	(3.42)	(3.78)	(3.99)	(2.86)	(2.68)
Sadness	-3.13	-1.37	-2.11	-1.48	-0.96
	(3.33)	(5.05)	(4.48)	(3.32)	(3.02)
**Production**					
	Principal Components				
	1 Mouth	2 Head displacement	3 Yaw	4 Velocity/ acceleration	5 Eyebrow
Happiness	11.89	8.61	9.91	6.52	5.78
	(11.15)	(13.29)	(13.47)	(8.93)	(7.50)
Irritation	8.11	3.81	4.84	3.18	2.91
	(8.20)	(8.32)	(9.96)	(5.75)	(5.02)
Neutral	1.63	-2.19	-1.85	-1.02	-0.31
	(5.77)	(4.91)	(4.80)	(3.57)	(3.60)
Sadness	3.67	2.60	1.77	1.49	0.57
	(7.54)	(8.31)	(7.17)	(5.71)	(3.72)
**Post-production**					
	Principal Components				
	1 Mouth	2 Head displacement	3 Yaw	4 Velocity/ acceleration	5 Eyebrow
Happiness	-0.90	2.63	2.40	1.86	-0.03
	(5.54)	(10.33)	(8.90)	(7.12)	(3.79)
Irritation	-4.19	-1.63	-1.95	-1.67	-1.57
	(3.09)	(4.55)	(4.85)	(3.04)	(2.66)
Neutral	-5.88	-5.07	-4.80	-3.33	-2.83
	(2.05)	(2.12)	(2.43)	(1.93)	(2.04)
Sadness	-4.78	-2.51	-2.80	-2.08	-2.06
	(3.24)	(5.34)	(5.06)	(3.64)	(2.71)

Table 3 (a-c)

Principal component scores by singer and epoch. (Standard deviations are in parentheses).

Pre-production

Singer	Components				
	1 Mouth	2 Head Disp.	3 Yaw	4 Head vel/acc	5 Eyebrow
1	-1.07	-2.69	-1.67	-0.80	0.52
	(3.77)	(2.62)	(3.09)	(2.36)	(3.00)
2	-3.38	-4.73	-4.50	-2.14	-1.65
	(3.30)	(1.97)	(2.17)	(2.02)	(2.25)
3	-1.36	0.06	-1.23	-0.88	-0.76
	(5.64)	(6.33)	(5.92)	(4.10)	(3.72)
4	0.75	2.31	2.18	1.33	3.56
	(4.80)	(5.28)	(5.51)	(3.64)	(4.51)
5	-2.64	-1.45	-2.00	-2.65	-1.74
	(3.30)	(3.78)	(3.94)	(1.82)	(2.35)
6	-2.79	1.87	0.56	1.17	-1.31
	(3.42)	(6.07)	(4.86)	(4.47)	(2.48)
7	-6.21	-6.31	-6.48	-4.69	-2.92
	(1.63)	(1.26)	(1.33)	(0.92)	(1.85)

Production

Singer	Components				
	1 Mouth	2 Head Disp.	3 Yaw	4 Head vel/acc	5 Eyebrow
1	4.18	-1.28	0.76	0.66	1.03
	(6.28)	(4.17)	(5.53)	(3.60)	(3.44)
2	4.63	-2.20	-1.45	0.31	0.55
	(6.70)	(3.21)	(3.84)	(3.13)	(3.22)
3	5.83	5.19	4.90	2.75	1.48
	(8.38)	(9.35)	(11.03)	(5.87)	(5.13)
4	13.09	14.02	13.83	9.49	8.74
	(11.48)	(14.14)	(14.76)	(9.08)	(8.52)
5	5.19	2.22	2.47	0.36	1.84
	(6.71)	(4.80)	(5.13)	(3.37)	(3.84)
6	11.09	7.22	7.46	6.28	2.81
	(10.69)	(10.96)	(11.13)	(8.56)	(4.71)
7	0.27	-2.74	-2.31	-2.04	-0.76
	(7.11)	(4.52)	(5.63)	(3.27)	(3.91)

Post-production

	Components				
Singer	1 Mouth	2<br>Head Disp.	3<br>Yaw	4<br>Head vel/acc	5<br>Eyebrow
1	-3.50	-1.83	-1.38	-0.64	-0.63
	(3.29)	(4.55)	(5.15)	(3.51)	(2.79)
2	-4.30	-4.59	-4.16	-1.79	-1.63
	(2.75)	(2.11)	(2.40)	(2.28)	(2.45)
3	-4.53	-2.03	-1.65	-1.78	-2.55
	(3.08)	(4.17)	(5.05)	(3.07)	(2.34)
4	-2.57	1.29	1.05	0.10	-0.43
	(3.96)	(6.55)	(6.36)	(4.28)	(3.31)
5	-5.92	-3.86	-4.38	-3.72	-3.79
	(2.16)	(2.72)	(2.49)	(1.56)	(1.40)
6	-0.48	5.13	3.97	3.33	0.40
	(6.57)	(12.49)	(10.29)	(8.45)	(3.82)
7	-6.25	-5.63	-5.96	-4.65	-2.74
	(2.51)	(2.13)	(2.17)	(1.13)	(2.36)
